# Supplementary material for: Development of a computer-aided design software for the quantitative evaluation of aesthetic damage
Source: PLoS One. 2019 Dec 18;14(12):e0226322. doi: 10.1371/journal.pone.0226322 (PMC6919621; doi:10.1371/journal.pone.0226322)
Supplement: S1 Code site — 1. (ZIP) [file pone.0226322.s001.zip › [EESC_jr][Projeto]Relatorio_07_21/reports/templates_zip/gentelella-master/vendors/nprogress/index.html]

NProgress: slim progress bars in JavaScript


# NProgress*.js*

A nanoscopic progress bar. Featuring realistic
trickle animations to convince your users that something is happening!

*NProgress***.start()**
—
shows the progress bar

*NProgress***.set(0.4)**
—
sets a percentage

*NProgress***.inc()**
—
increments by a little

*NProgress***.done()**
—
completes the progress

Download
v

Perfect for Turbolinks, Pjax, and other Ajax-heavy apps.

HN
